# The Microenvironment of Small Intestinal Neuroendocrine Tumours Contains Lymphocytes Capable of Recognition and Activation after Expansion

**DOI:** 10.3390/cancers13174305

**Published:** 2021-08-26

**Authors:** Tobias Hofving, Frank Liang, Joakim Karlsson, Ulf Yrlid, Jonas A. Nilsson, Ola Nilsson, Lisa M. Nilsson

**Affiliations:** 1 Sahlgrenska Center for Cancer Research, Department of Pathology and Genetics, Institute of Biomedicine, University of Gothenburg, 40530 Gothenburg, Sweden; tobias.hofving@gu.se (T.H.); ola.nilsson@llcr.med.gu.se (O.N.); 2 Department of Microbiology and Immunology, Institute of Biomedicine, University of Gothenburg, 40530 Gothenburg, Sweden; frank.liang@gu.se (F.L.); ulf.yrlid@gu.se (U.Y.); 3 Sahlgrenska Translational Melanoma Group, Sahlgrenska Center for Cancer Research, Department of Surgery, Institute of Clinical Sciences, Sahlgrenska University Hospital, University of Gothenburg, 40530 Gothenburg, Sweden; joakim.karlsson@perkins.org.au (J.K.); lisa.nilsson@perkins.org.au (L.M.N.); 4 Harry Perkins Institute of Medical Research, UWA Centre for Medical Research, University of Western Australia, 6 Verdun Street, Nedlands, WA 6009, Australia

**Keywords:** small-intestinal neuroencocrine tumors, neuroendocrine tumours, patient-derived xenografts, tumour-infiltrating lymphocytes

## Abstract

**Simple Summary:**

The body‘s immune system can recognize tumors because they often contain proteins that are either different from or more abundant than in normal cells. Here, we characterised the immune cells of a rare tumor type called small-intestinal neuroendocrine tumors (SINET). We find that so called tumour-infiltrating lymphocytes (TILs) can be grown in the laboratory and activated by challenging them with digested tumor. This study provides insights into the largely unknown SINET immune landscape and reveals the anti-tumour reactivity of TILs, which might merit adoptive T cell transfer as a feasible treatment option for patients with SINET.

**Abstract:**

Traditionally, immune evasion and immunotherapy have been studied in cancers with a high mutational load such as melanoma or lung cancer. In contrast, small intestinal neuroendocrine tumours (SINETs) present a low frequency of somatic mutations and are described as genetically stable tumours, rendering immunotherapies largely unchartered waters for SINET patients. SINETs frequently metastasise to the regional lymph nodes and liver at the time of diagnosis, and no curative treatments are currently available for patients with disseminated disease. Here, we characterised the immune landscape of SINET and demonstrated that tumour-infiltrating lymphocytes (TILs) can be expanded and activated during autologous tumour challenge. The composition of lymphocyte subsets was determined by immunophenotyping of the SINET microenvironment in one hepatic and six lymph node metastases. TILs from these metastases were successfully grown out, enabling immunophenotyping and assessment of PD-1 expression. Expansion of the TILs and exposure to autologous tumour cells in vitro resulted in increased T lymphocyte degranulation. This study provides insights into the largely unknown SINET immune landscape and reveals the anti-tumour reactivity of TILs, which might merit adoptive T cell transfer as a feasible treatment option for patients with SINET.

## 1. Introduction

The immune system monitors the body for foreign antigens but can also detect cancer cells. Mutated proteins (neoantigens) or highly overexpressed proteins (tumour-associated antigens) give rise to peptides presented on MHC molecules on, e.g., cancer cells, which can be recognised by antigen-specific T lymphocytes [1]. However, cancer cells utilise several immune evasion mechanisms, most notably expression of PD-L1, the ligand of the immune checkpoint PD-1. This is the basis of immune checkpoint inhibitor (ICI) therapy which, along with adoptive T cell therapy (ACT), has demonstrated curative effects in many tumour types [2,3].

Resistance to immunotherapies can occur by many different mechanisms including expression of immune checkpoint markers and downregulation of the antigen presentation machinery. Furthermore, some tumour types such as small intestinal neuroendocrine tumours (SINET) do not carry a high mutational burden and hence present fewer neoantigens [4,5]. Combined with a promotion of an immunosuppressive microenvironment seen in neuroendocrine neoplasms [6] and other tumour types, this results in a poor expansion of antigen-specific T cells and immune evasion.

Patient-derived xenograft models are mouse models generated by transplanting tumour cells or tissues directly from patients most often without prior in vitro cultivation. They are generally regarded as more accurate models than cell line-derived xenografts since they grow slower, display a similar histopathology architecture, represent the genetic heterogeneity of patients, respond to treatments more similarly to patients, and can even be used as mouse models for precision oncology treatment decisions, so-called mouse avatars [7,8,9,10]. Nevertheless, these models have a caveat in being immunocompromised, which is a pre-requisite to grow human cells in mice. Attempts at immune humanisation of xenograft models have been conducted so that PDX models can be used for immunotherapy studies, but these models are rarely autologous [11,12]. We previously developed the PDX version 2 (PDXv2) model, where melanoma tumours were grown in immunocompromised non-obese diabetic severe combined immunodeficiency interleukin 2 receptor gamma knockout mice (NSG/NOG) transgenic for human interleukin 2 (hIL2-NOG) [13]. In PDXv2 mice generated from a subset of patients, eradication of autologous cancer cells can be achieved by injecting TILs, which is accompanied by in vivo expansion of the TILs.

Currently, no genetically engineered mouse model exists for SINET, and only a few cell lines have been described to establish xenograft mouse models [14]. In fact, some cell lines previously assumed as SINET were in fact EBV-transformed lymphoblastoid cell lines [15]. The aim of this study was to characterise the genetics and immune landscape of SINET by RNA/exome sequencing and flow cytometry and develop methods to study T cell potency and reactivity in vitro and in vivo.

## 2. Results

### 2.1. Exome and RNA Sequencing of Metastatic SINET Reveals Insight into Genetics and Immunology

**Figure 1:**
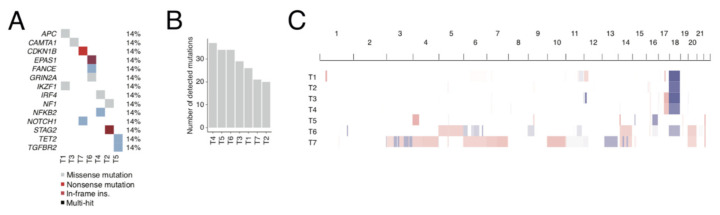
Genomic alterations. (**A**) Somatic mutations in known cancer genes. (**B**) Total number of detected non-synonymous somatic mutations in each sample. (**C**) DNA copy number changes. Blue indicates loss, while red indicates gain. The intensity of the respective colours reflects both the number of copies gained or lost and tumour purity.

We molecularly profiled six SINET lymph node metastases and one hepatic metastasis (Table 1) using exome and RNA sequencing. Eosin and haematoxylin staining revealed that the specimens consisted of tumour and stroma cells which had displaced the lymph node tissue (Supplementary Figure S1). Whole-exome and RNA sequencing revealed an average of 25 protein-modifying mutations per tumour (range: 45–69) (Figure 1A). Of these mutations, 14 were listed in COSMIC Cancer Gene Census (CCGC, Figure 1B), all of which were only present in one patient. While SINETs contain relatively few somatic mutations, they also harbour several recurrent chromosomal aberrations [16,17,18]. Tumours can be sub-grouped based on chromosomal aberrations into one larger group characterised by chromosomal losses, notably loss of chromosome 18, and a smaller group characterised by multiple chromosomal gains, notably 4, 5, 7, and 10 [17]. Five out of seven tumours (T1, T2, T3, T4, and T6) contained loss of chromosome 18, one tumour (T7) had gains of multiple characteristic chromosomes, and one tumour (T5) belonged to neither of these groups (Figure 1C).

**Figure 2:**
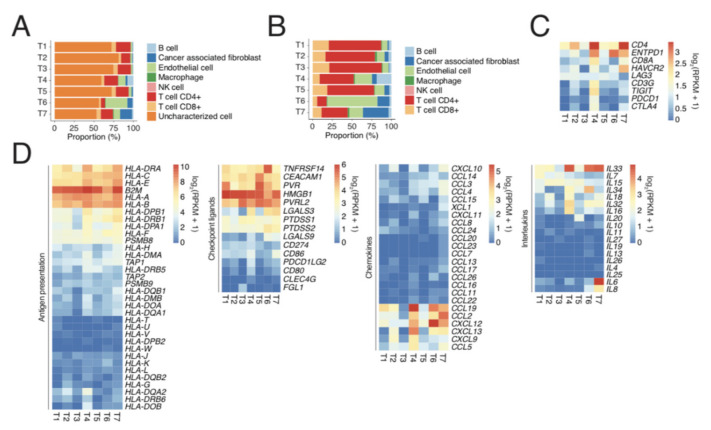
Immune profiling using RNA-seq. (**A**) Inferred proportions of constituent cell types in bulk samples, based on deconvolution using EPIC. (**B**) Same as (**A**), but after removing uncharacterised cells (most likely cancer cells). (**C**) Expression levels of T cell markers and checkpoint receptors. (**D**) Expression levels of genes involved in antigen presentation, immune checkpoint ligands, chemokines, and interleukins.

We used the CIBERSORT script on RNA sequencing data to gain insight into the presence of immune cells in the SINETs. The major cell type in the tumours was the “uncharacterised” tumour cells (Figure 2A). Focusing on the characterised cells, CD4+ T cells were the most common cell type in the tumours (Figure 2B). Immune checkpoint proteins PD-1 and TIM-3 were expressed primarily in the samples which had the most CD8+ cells (Figure 2C). Both HLA class 1/2 and immune checkpoint ligands for TIM-3 (e.g., HMGB) and TIGIT (e.g., PVR and PVRL2) were highly expressed by most tumours (Figure 2D). However, cytokine/chemokine expression was more variable.

### 2.2. Single-Cell Analyses by IHC and Flow Cytometry of Tumours and TILs Show Immune Heterogeneity

**Figure 3:**
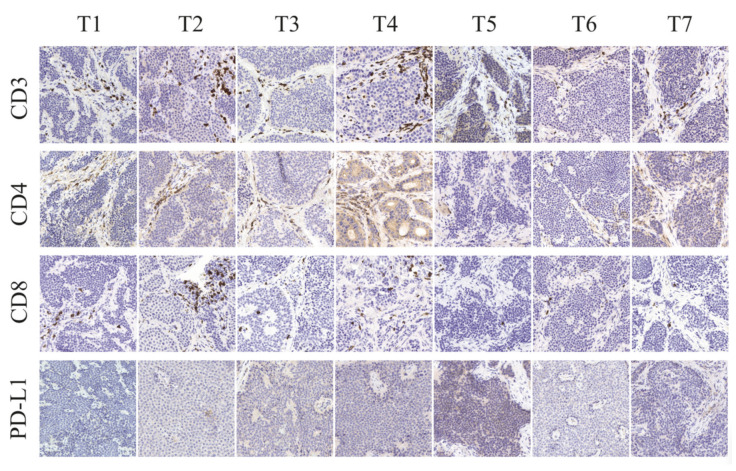
Localisation and distribution of immune cell subsets in small intestinal neuroendocrine tumours. CD3+ T lymphocytes, CD4+ T lymphocytes, CD8+ T lymphocytes, NKp46+ NK cells, and expression of PD-L1 were evaluated on seven small intestinal neuroendocrine tumours (T1−T7) using immunohistochemistry.

CD3+ T lymphocytes were present in all tumours, and more than 90% were localised to the tumour stroma and the interphase between the tumour stroma and tumour nests (Figure 3). A minority of the CD3+ T lymphocytes had infiltrated the tumour nests and were located intra-tumourally. The total amount of CD3+ T lymphocytes varied between tumours of different patients, where tumours T5 and T6 had notably fewer CD3+ cells compared to the other tumours (Figure 3). Staining of CD4+ T lymphocytes (T helper) and CD8+ T lymphocytes (cytotoxic T cells) on serial sections revealed that the localisation of CD4+ and CD8+ T lymphocytes frequently overlapped. CD4+ T lymphocytes and CD8+ T lymphocytes were found in the stroma, interphase, and intra-tumourally, but CD8+ T lymphocytes were more frequently localised intra-tumourally (Supplementary Figure S2). Interestingly, very few NKp46+ NK cells were observed (<10 cells/full tumour section), and all resided within the tumour stroma. In the tumours, several areas of T lymphocyte aggregation were observed. These were not associated with any morphological features observable by eosin and haematoxylin staining and did not express granzyme B to a larger extent compared to other T lymphocytes. As immune cells would normally eliminate abnormal cells such as cancer cells, they need to adapt and, as such, circumvent immune cell recognition and/or activation. The recently successful PD-1:PD-L1 checkpoint inhibitors demonstrated the potency of abrogating this inhibition. Previous studies have varied largely in methods and reported ranges investigating the PD-L1 and PD-L2 positivity of SINETs using immunohistochemistry. Reports of PD-L1 positivity ranged between 0 and 39%, and for PD-L2, between 0 and 82% [19,20,21]. Here, using the FDA-approved PD-L1 staining kit, we were only able to detect weak PD-L1 expression in the T2 tumour (Figure 3).

**Figure 4:**
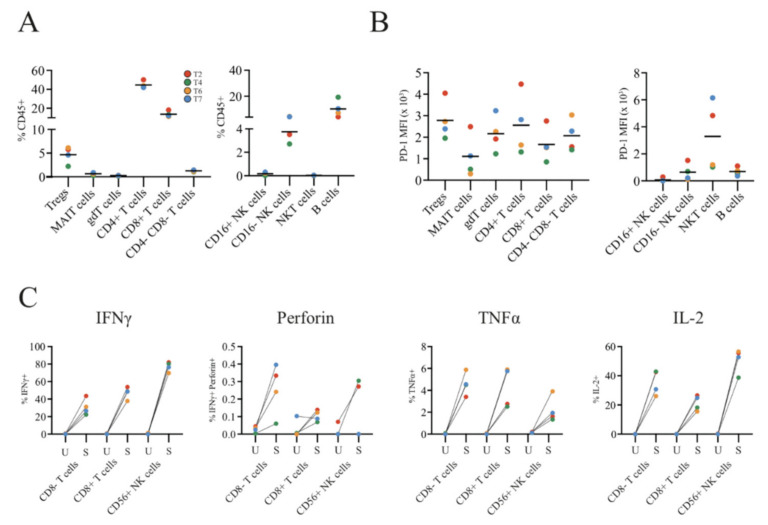
The immune microenvironment of small intestinal neuroendocrine tumours. (**A**) The immune microenvironment of four small intestinal neuroendocrine tumours was characterised using flow cytometry after staining with a lymphocyte antibody panel. The immune microenvironment mostly contained CD4+ T lymphocytes, CD8+ T lymphocytes, and B cells, but also many other immune subsets. (**B**) Most immune subsets expressed PD-1 in all four patient tumours. (**C**) To assess the viability and functionality of the tumour-infiltrating lymphocytes, we stimulated them with PMA and ionomycin and could observe a marked increase in IFNγ, perforin, TNF-α, and IL-2 in CD8+ T lymphocytes, non-CD8+ T lymphocytes, and CD56+ NK cells. U, unstimulated; S, stimulated.

To characterise and properly quantify SINET-infiltrating lymphocytes, we performed a flow cytometry analysis of single-cell suspensions of SINET with a comprehensive lymphocyte panel on four patient tumours (T2, T4, T6, and T7). We were able to identify several immune subsets, including CD4+ T cells, CD8+ T cells, regulatory T cells (Tregs), MAIT cells, γδ T cells, CD16+ NK cells, CD16- NK cells, NKT cells, and B cells (Figure 4A). The most abundant population within viable CD45+ immune cells was CD4+ T lymphocytes, followed by CD8+ T lymphocytes, and B cells. CD56+ CD16- NK cells and Tregs belonged to the minor populations, and low levels of MAIT cells, γδ T cells, and the undefined CD4-CD8- cells were also identified. Interestingly, the proportion of various immune subsets was strikingly similar in all four tumours. PD-1 was expressed in all immune subsets but had a high variability between tumour samples (Figure 4B). To investigate whether infiltrating T lymphocytes were capable of cytokine responses, we stimulated SINET suspensions with phorbol myristate acetate (PMA) and ionomycin. This resulted in a potent increase in interferon-γ, perforin, tumour necrosis factor α, and interleukin-2 (IL-2) in CD8+, non-CD8-positive (CD4+) T cells, and CD56+ NK cells (Figure 4C). Furthermore, although most CD4+/CD8+ T lymphocytes and NK cells contained only one of the four cytokines, polyfunctionality was still observed in a considerable proportion of T lymphocytes and NK cells (Supplementary Figure S3). In particular, TILs from tumours T6 and T7 had higher polyfunctionality of both CD4+ and CD8+ T lymphocytes than TILs from the T3 and T4 tumours.

**Figure 5:**
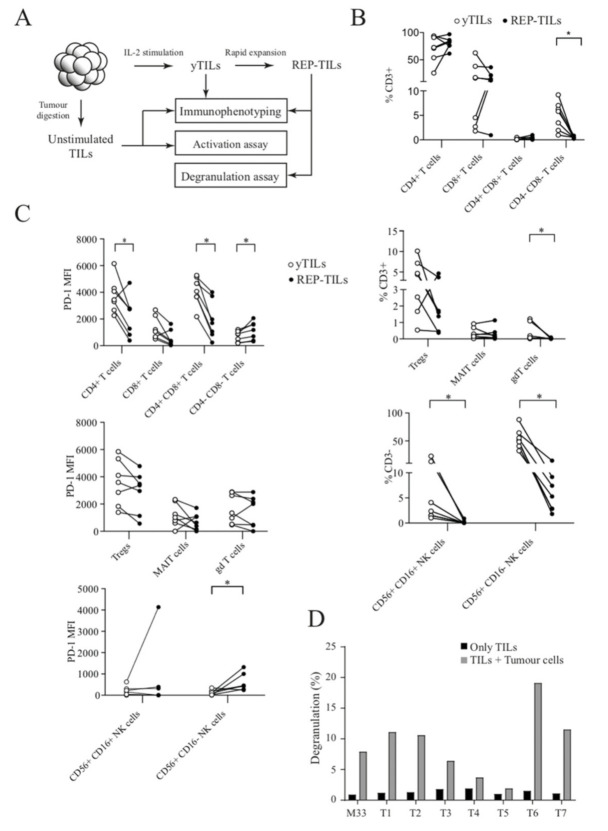
Generation and characterisation of yTILs and REP-TILs. (**A**) In addition to immunophenotyping and activation assay performed on unstimulated TILs, yTILs were generated by IL-2 stimulation and REP-TILs by subsequent rapid expansion protocol. Both yTILs and REP-TILs were immunophenotyped, and in addition, the degranulation of REP-TILs was measured in response to autologous tumour cells. (**B**,**C**) During the expansion of yTILs to REP-TILs, the proportion of various immune subsets changed. Notably, the proportion of MAIT cells and both CD16+ and CD16- NK cells decreased among CD3+ lymphocytes (**B**). PD-1 expression decreased in most immune subsets during TIL expansion (**C**,**D**). REP-TILs generated from SINETs degranulate in response to autologous tumour cells, measured as increased surface positivity of the T cell granule marker CD107a. TILs and tumour cells from a malignant melanoma patient (MM33) were used as a positive control (* *p* < 0.05, Wilcoxon test).

It has been shown that immunologic inhibition of TILs can be overcome by the presence of exogenous interleukin-2 (IL-2) [22,23]. Such activated TILs can be made into large quantities by rapid expansion, as used for ACT therapy to treat patient tumours in the clinic [24]. After characterising unstimulated TILs with immunophenotyping and an activation assay, we next attempted to characterise TILs generated through IL-2 stimulation (young TILs, yTILs) and the rapid expansion protocol (REP-TILs) by immunophenotyping and a degranulation assay (Figure 5A). The yTILs were generated by culture of excised tumour tissue collected from surgery in media supplemented with IL-2. TILs that left the tumour were observed in these cultures within 24 h, and these TILs gave rise to the yTILs harvested after 21−28 days. To generate REP-TILs, yTILs were co-cultured with irradiated peripheral blood mononuclear cells and stimulated with anti-CD3 antibody and exogenous IL-2 for 14 days. We successfully expanded yTILs of all tumours starting from 5 × 10^4^ lymphocytes to an average of 6.6 × 10^7^ lymphocytes (range 3.6 × 10^7^–9.0 × 10^7^). The amount of immune cell subpopulations can be predictive for the clinical response to ACT. For example, the number of CD8+CD27+ cells injected has been shown to be associated with the objective response [2]. The proportions of both CD4+ T lymphocytes and CD8+ lymphocytes within the obtained REP-TILs were altered (Figure 5B). Notably, a large increase in CD4+ T lymphocytes was observed for tumour T4 (25.7% to 76.8%) and in CD8+ T lymphocytes in tumours T2 (4.5% to 21.4%) and T3 (2.7% to 11.9%). The proportion of CD4+CD8+ T lymphocytes remained largely unchanged, and the CD4-CD8- cells decreased. The frequency of Tregs decreased in all patients, except for one patient. Whereas the levels of MAIT cells were largely unchanged, the γδT cells decreased. Both the proportion of CD56+CD16+ and CD56+CD16- NK cells decreased drastically. Overall, PD-1 expression was downregulated on REP-TILs, except for the CD4-CD8- population where PD-1 was induced instead (Figure 5C). Interestingly, PD-1 was also upregulated on CD56+CD16- NK cells by the REP protocol.

We cultured REP-TILs with single-cell suspensions of autologous tumour cells and assessed T cell granulation by using anti-CD107a (LAMP1). CD107a is normally expressed on the internal surface of lysosomes and granules and exposed when lymphocytes degranulate in response to stimuli [25]. We used REP-TILs from a malignant melanoma patient as a positive control since these TILs have previously been demonstrated to be reactive against autologous tumour cells in vivo [13]. The REP-TILs from all SINETs degranulated more when tumour cells were present (Figure 5D), indicating that the SINET REP-TILs can recognise and respond to autologous tumours. The amount of degranulation differed substantially between tumour samples. REP-TILs from tumours T1, T2, T6, and T7 degranulated more than REP-TILs from melanoma, while TILs from tumours T4 and T5 degranulated to a lesser degree.

### 2.3. SINET Cells Can Survive in Long-Term Tumour Grafts but Are Resistant to T Cell Killing In Vivo

To establish PDX models of SINET, we transplanted a total of 38 pieces of surgically resected tumours from 36 patients into 55 NOG mice. Since no SINET PDX has previously been successfully established directly from patient biopsies, and the take-rate of neuroendocrine tumours overall seems poor [26], we tried xenografting the tumours both from cryopreserved material and from fresh tumour tissue obtained at surgery. Both subcutaneous transplantation and injection of tumour cells from a hepatic metastasis into the mouse liver were performed (Table 2). One tumour from a grade 1 liver metastasis that was transplanted subcutaneously was successfully propagated and grown through two passages (Supplementary Figure S4A). However, of the 54 mice transplanted, a majority had to be sacrificed for ethical reasons, mainly due to old age, without any observed tumour growth. Autopsy revealed no tumours in any of the sacrificed tumour-free mice. Immunohistochemistry did, however, reveal a small amount of tumour cells expressing SINET markers, a minority of which also expressed proliferation marker Ki67, in those mice that had tumours that had not grown but still remained palpable after original tumour implantation.

To further study this model, we applied our recently established protocol and transplanted new tumour pieces from three patients into either ordinary NOG/NSG mice or into hIL2-NOG mice [27]. After three weeks, mice were sacrificed, and tumour biopsies were collected for immunohistochemistry. In line with previous attempts at generating PDX models, all the biopsies were necrotic, but a few live SINET cells were detected using anti-synaptophysin staining (Supplementary Figure S4B). However, there was no noticeable growth suppression in hIL2-NOG mice compared to tumours grown in NOG mice (Supplementary Figure S4C). This was not due to a lack of T cells, as they were detected in the tumours and blood of some hIL2-NOG mice (Supplementary Figure S4B,D). In fact, T cell expansion probably contributed to the slightly enhanced tumour size in the hIL2-NOG mice (Supplementary Figure S4C) akin to the phenomenon known as pseudo-progression, which occasionally occurs in cancer patients treated with immunotherapy. The expansion of T cells was also measurable in the blood using granzyme B ELISA (Supplementary Figure S4E), suggesting that some of the T cells were reactive.

## 3. Material and Methods

### 3.1. Whole-Exome Sequencing

After informed consent, snap-frozen biopsies were collected at surgery of patients diagnosed with SINET. DNA was extracted using AllPrep DNA/RNA mini kit (Qiagen, Hilden, Germany). Exome sequencing was performed at the GeneCore SU core facility. Raw reads were mapped to the human genome, and mutations and copy number alterations were assessed using the GATK and Mutect2 R packages.

### 3.2. Immunohistochemistry

Paraffin-embedded tissue blocks from patient tumours prepared for routine clinical histopathology were obtained from Sahlgrenska University Hospital. Sections (3–4  μm) from paraffin blocks were placed on glass slides and treated in Dako PT-Link using EnVision™ FLEX Target Retrieval Solution (high pH). The following primary antibodies were used: anti-CD3 (Dako/Agilent Technologies, Santa Clara, CA, USA; IR503), anti-CD4 (Dako; 4B12), anti-CD8 (Dako; C8/144B), anti-NKp46 (R&D Systems, Minneapolis, MN, USA; 195314), anti-granzyme B (Novocastra/Leica Biosystems, Wetzlar, Germany; 11F1), anti-PD-L1 kit (Dako; PD-L1 IHC 28-8 pharmDx; according to manufacturer’s instructions), and anti-Ki-67 (Dako; MIB-1). Immunohistochemical staining was performed in a Dako Autostainer Link using EnVision™ FLEX according to the manufacturer’s instructions (DakoCytomation). EnVision™ FLEX+ (LINKER) mouse was used for anti-NKp46, anti-CD25, anti-granzyme B, and anti-Ki-67. Positive and negative controls were included in each run. Each staining was evaluated by a board-certified pathologist (O.N.). The Ki67 index was calculated by manually counting the percentage of labelled tumour cell nuclei on printed images [28].

### 3.3. Generation of Tumour-Infiltrating Immune Cells

Patient tumour tissue samples were obtained from patients undergoing surgery for SINET disease at Sahlgrenska University Hospital, Gothenburg, Sweden. Unstimulated TILs for characterisation and stimulation experiments were generated from 1–2 mm^2^ cryopreserved tumour pieces that were incubated with 2 mg/mL collagenase type I (C-0130, Sigma-Aldrich, St. Louis, MO, USA) and >5 ng/mL deoxyribonuclease I (D-4263, Sigma-Aldrich) at 37 °C in 5% CO_2_ for 2 h. The resulting single-cell suspension was filtered and washed with PBS. yTILs were generated by cutting tumour tissue obtained directly from surgery into 1–2 mm^2^ pieces that were placed in separate wells in a 24-well plate (Sarstedt, Newton, NC, USA) with 2 mL of culture medium (90% RPMI 1640 (Invitrogen, Waltham, MA, USA), 10% heat-inactivated human AB serum (HS, Sigma-Aldrich), and 6000 IU/mL recombinant human IL-2 (Peprotech) and gentamicin (Invitrogen). Young TIL (yTIL) cultures were obtained by pooling TILs from each fragment as previously described [29,30,31], before being cryopreserved. To generate REP-TILs, yTILs were expanded using a standard small-scale REP [31]. In short, irradiated (40 Gy) allogeneic feeder cells (5 × 10^6^), 30 ng/mL anti-CD3 antibody (Miltenyi, Bergisch Gladbach, Germany; OKT3), 5 mL culture medium, 5 mL REP medium (AIM-V, Invitrogen) supplemented with 10% HS, and 6000 IU/mL IL-2 and yTILs (5 × 10^4^) were mixed in a 25 cm^2^ tissue culture flask. Flasks were incubated upright at 37 °C in 5% CO_2_. On day 5, half of the medium was replaced. On day 7 and every day thereafter, cells were split into further flasks with additional REP medium as needed to maintain cell densities around 1–2 × 10^6^ cells/mL. On days 10–14, cells were harvested and cryopreserved.

### 3.4. Characterisation and Stimulation of Tumour-Infiltrating Immune Cells

Filtered single-cell suspensions were washed with PBS and were either stained directly for characterisation of immune cell subsets or resuspended in complete media: RPMI 1640 GlutaMAX^TM^ supplemented with 10% fetal calf serum, 1% HEPES, 0.1% gentamicin, and 1% penicillin-streptomycin (Gibco) for overnight stimulation with phorbol myristate acetate (0.01 µg) and ionomycin (0.25 µg) in presence of brefeldin A (5 µg, Sigma) at 37 °C in 5% CO_2_. Surface markers were stained as previously described [32] with a cocktail of fluorescent monoclonal antibodies (mAbs): anti-CD127, CD19, CD45, γδTcR, Vα24 TcR, Vα7.2 TcR (Biolegend, San Diego, CA, USA), CD11c, CD15, CD16, CD25, CD3, CD4, CD56, CD8, PD-1, and HLA-DR (BD). Zombie Red (Biolegend) or Aqua (Invitrogen) fixable viability kits were used for dead cell detection. For intracellular staining, surface-stained cells were treated with fixation/permeabilisation kit (BD) and stained with anti-IFNγ, perforin (BD), TNFα, and IL-2 (Biolegend) mAbs. Analysis was performed using FlowJo software v.9.9.6 (Tree Star). Polyfunctional T cell cytokine responses were assessed using SPICE software v.5.35 (freely available and downloaded 22nd Oct. 2018 from http://exon.niaid.nih.gov/spice/). Statistical analysis was performed using non-parametric paired comparison with Wilcoxon test in GraphPad Prism software (San Diego, CA, USA) and considered significant at p* < 0.05.

### 3.5. Degranulation Assay

Tumour single-cell suspensions were generated by incubating tumour pieces with 2 mg/mL collagenase type I (C-0130, Sigma-Aldrich) and >5 ng/mL deoxyribonuclease I (D-4263, Sigma-Aldrich) at 37 °C in 5% CO_2_ for 2 h. REP-TILs generated from the patient tumours were thawed, and 3 × 10^5^ cells were transferred to two wells on a cone-bottomed 96-well plate (Sarstedt) in RPMI 1640 GlutaMAX^TM^ supplemented with 10% heat-inactivated human serum (HS, Sigma-Aldrich) and 50 ng/mL gentamicin. APC-CD107a antibody (BD; clone H4A3) was added to both wells, and to one well, 10^5^ autologous cancer cells were added. After incubation at 37 °C in 5% CO_2_ for 4 h, cells were harvested in MACS-buffer, strained, and analysed using a BD Accuri C6 Plus flow cytometer with associated software (v.1.0.23.1).

### 3.6. Patient-Derived Xenografts

SINET surgical specimens were obtained from patients undergoing surgery at Sahlgrenska University Hospital, Gothenburg, Sweden. Tumours were xenografted to 6−15-week-old immunocompromised, non-obese severe combined immune-deficient interleukin-2 chain receptor γ knockout mice (NOG mice; Taconic) or NOG mice transgenic for human IL-2 (hIL2-NOG; Taconics). For subcutaneous transplantations, tumour tissue was cut into 1–2 mm^2^ pieces and either transplanted directly from surgery or after cryopreservation into the flank of the mouse. Orthotopic transplantations were performed with liver metastasis tumour pieces that had been incubated with 2 mg/mL collagenase type I (C-0130, Sigma-Aldrich) and >5 ng/mL deoxyribonuclease I (D-4263, Sigma-Aldrich) at 37 °C in 5% CO_2_ for 2 h, before being cryopreserved. Liver metastasis single-cell suspensions were injected into the mouse liver. When mice were sacrificed, autopsy was performed to validate any lack of tumour growth.

### 3.7. Preprocessing of RNA-Seq Data

RNA-seq reads were aligned to the 1000 Genomes [33] version of the hg19 human reference genome (v.37) with STAR (v.2.7.1a) [34]. Arguments used were “--twopassMode Basic --outFilterType BySJout --sjdbOverhang 75 --outSAMmapqUnique 60 --outSAMstrandField intronMotif --outSAMunmapped Within --outReadsUnmapped None --chimSegmentMin 12 --chimJunctionOverhangMin 8 --chimOutJunctionFormat 1 --alignSJDBoverhangMin 10 --alignMatesGapMax 100000 --alignIntronMax 100000 --alignSJstitchMismatchNmax 5 -1 5 5 --chimMultimapScoreRange 3 --chimScoreJunctionNonGTAG -4 --chimMultimapNmax 20 --chimNonchimScoreDropMin 10 --peOverlapNbasesMin 12 --peOverlapMMp 0.1 --alignInsertionFlush Right --alignSplicedMateMapLminOverLmate 0 --alignSplicedMateMapLmin 30”. Known splice junctions from the NCBI GRCh37.75 reference genome annotation were also provided as input. Read counts per gene were then obtained from these alignments using htseq-count (HTSeq v. 0.11.2) [35], with the arguments “-r name -q -f bam -s reverse -m intersection-strict”, relative to the NCBI GRCh37.75 reference genome annotation.

### 3.8. Immune Cell Deconvolution

To determine cell types contributing to the composition of bulk RNA-seq samples, RPKM normalised gene expression values were used as input to the function *deconvolute* from the R package immunedeconv (v. 2.0.3), which contains wrapper functions to run a number of different cell type deconvolution methods. The parameter “method = ‘epic’” was used to run EPIC (v. 1.1.5) [10].

### 3.9. Preprocessing of Exome Sequencing Data

Exome sequencing reads were aligned to the 1000 Genomes version of the hg19 human reference genome (v. 37) with bwa [4] (v. 0.7.17) using the arguments “mem -t 10 -M -R”. Alignments corresponding to multiple sequencing runs of the same sample were merged using the samtools “merge” command (v. 1.9). Duplicate reads were marked with MarkDuplicates (GATK v. 4.1.3.0) [5] using default parameters. Base quality score recalibration was performed with BaseRecalibrator and ApplyBQSR (GATK) in two passes using the same reference genome as well as lists of known polymorphisms from the GATK resource bundle (files “dbsnp_138.b37.vcf”, “1000G_phase1.indels.b37.vcf”, and “Mills_and_1000G_gold_standard.indels.b37.vcf”).

### 3.10. Mutation Calling

Variant calling for exome sequencing alignments was performed with Mutect 2 [6] (GATK v. 4.1.3.0) using the parameters “—genotype-germline-sites true —genotype-pon-sites true —af-of-alleles-not-in-resource 0.0000025 —disable-read-filter MateOnSameContigOrNoMappedMateReadFilter”. The GnomAD [7] population variant database was provided as a germline resource, together with the same reference genome as above. The analysis was restricted to exome target regions corresponding to Agilent SureSelect Clinical Research Exome v2. Variant qualities were further assessed using FilterMutectCalls (GATK). These variants were then annotated using the script vcf2maf.pl (https://github.com/mskcc/vcf2maf, accessed on 2 May 2019), which relies on VEP, using the v. 98 build of the VEP reference database for the GRCh37 genome. Variants were further filtered using custom scripts to remove genes with >0.001 frequency in GnomAD, ExAC, and genes with dbSNP identifiers, unless any of these variants were whitelisted. Variants were whitelisted if they were either listed as oncogenes in Cancer Gene Census (CGC) and the exact mutation was listed in COSMIC or if they were listed as tumour suppressors in CGC. The resulting list was further filtered to remove variants that only occurred in more than one PDX sample but not in any patient biopsy.

### 3.11. Copy Number Analysis

Copy number segmentation was performed with CNVkit (v. 0.9.6), by first running the “cnvkit.py batch” command with matching tumour and normal files, exome target regions based on Agilent SureSelect Clinical Research Exome V2, the 1000 Genomes version of the hg19 human reference genome (v. 37), and a list of problematic regions to exclude (http://hgdownload.cse.ucsc.edu/goldenpath/hg19/encodeDCC/wgEncodeMapability/wgEncodeDukeMapabilityRegionsExcludable.bed.gz). The resulting output was converted to SEG-formatted files using the commands “cnvkit.py segmetrics” (parameters: “—ci -a 0.05”) followed by “cnvkit.py call” (parameters: “—center “median” —purity 1 —filter ci”) and “cnvkit.py export seg”.

## 4. Conclusions

Here, we characterised and quantified the lymphocyte subsets in the hitherto much under-studied SINET immune microenvironment by RNA sequencing, flow cytometry, and immunohistochemistry. We showed that TILs from SINETs can be expanded, and the expanded REP-TILs elicit an anti-tumour response when challenged with autologous tumour cells. The latter finding implies that expanded TILs not only can detect the tumour cells but can also respond against them. This ability of SINET REP-TILs to recognise autologous tumours is in line with a previous report on circulating CD8+ T lymphocytes of SINET patients with specificity for SINET-associated peptides [36]. The fact that T lymphocytes directed against SINET-associated antigens when expanded could elicit an anti-tumour response suggests that TIL expansion can activate the immune cells. This effect is also observed when successfully treating cancer patients with expanded T lymphocytes in ACT.

Clinical responses to ACT can be modelled using transplanted PDX tumours and autologous T cells in human IL-2 transgenic NOG mice [13]. To evaluate whether ACT would be relevant for SINET patients, we sought to answer whether TILs can recognise, become activated, and eradicate tumours in vivo. Unfortunately, we were not able to generate PDX models from more than one patient biopsy. Although immunohistology verified the authenticity of our SINET model, serial transplantation did not result in new PDX models. Instead, we resorted to establishing tumour explant models in NOG or hIL2-NOG models, as previously described [27]. Our previous study on melanoma demonstrated that tumour growth in hIL2-NOG mice and in NOG correlates with poor survival following anti-PD1 ICI treatment of the corresponding patient. When establishing explant modes for SINET, we observed expansion of TILs in the hIL2-NOG mice, resulting in an expansion of the tumour size. However, only few SINET cells survived, even in NOG mice, meaning we were not able to assess if the injected TILs had tumour reactive capacity in vivo. Nevertheless, the ex vivo experiments do suggest that TILs from SINET can degranulate in the presence of autologous tumour digests. It is therefore likely that ACT with TILs might be effective in some patients with SINET. In melanoma, we found that a large fraction of CD8+ T cells predict TIL responses in hIL2-NOG PDXv2 mice and patients [13]. This may be a major limitation for SINET patients, since most of the T cells present in SINET are CD4+ T cells. On the other hand, the role of cytotoxic CD4+ TILs being able to cause durable responses in patients is being recognised [37]. Therefore, until proven otherwise, we cannot exclude that ACT therapy would work for patients with tumours that predominantly contain CD4+ TILs. This will be important to explore, since SINET still remains as a disease with few therapeutic options.

## Figures and Tables

**Table 1:** Clincopathological characteristics of patients and their small intestinal neuroendocrine tumours.

Patient ID	Gender	Age at Surgery	Disease Stage	Dead/Alive	Tumour ID	Tumour Metastasis Site	Tumour Grade (% Ki67)
P1	M	79	IIIB	AWD	T1	LN	G1 (1.5)
P2	F	69	IIIB	AWD	T2	LN	G1 (0.5)
P3	M	76	IV	AWD	T3	Hepatic	G1 (2.6)
			IV	AWD	T4	LN	G2 (3.7)
P4	M	57	IV	AWD	T5	LN	G1 (0.9)
P5	M	84	IV	AWD	T6	LN	G1 (1.3)
P6	M	75	IV	AWD	T7	LN	G2 (3.3)

Abbreviations: M, Male; F, Female; AWD: Alive with disease, LN: Lymph node metastasis.

**Table 2:** Take-rate of small intestinal neuroendocrine transplanted to NOG mice.

Transplantation Site	Subcutaneous		Orthotopic
Patient Tumour Site	Lymph Node	Hepatic	Hepatic
From surgery	0/15	1/4	NA
From cryofrozen	0/14	0/5	0/16
Total	0/29	1/8	0/16

Abbreviations: NA, Not available.

## Data Availability

RNA sequencing data are available under restrictions of controlled access at European Genome-Phenome Archive at accession EGAS00001003358 and any other data is available from the authors upon reasonable request.

## Supplementary Materials

The following are available online at https://www.mdpi.com/article/10.3390/cancers13174305/s1, Figure S1: Eosin & haematoxylin (H&E) staining of seven small intestinal neuroendocrine tumours used in the characterisation of the immune landscape., Figure S2: Localisation of CD8+ T lymphocytes., Figure S3: Polyfunctionality of CD8+ T lymphocytes, CD8- lymphocytes, and CD56+ NK cells., Figure S4: Establishment of small intestinal neuroendocrine tumour patient-derived xenografts.
