# Supplementary material for: The Microenvironment of Small Intestinal Neuroendocrine Tumours Contains Lymphocytes Capable of Recognition and Activation after Expansion
Source: Cancers (Basel). 2021 Aug 26;13(17):4305. doi: 10.3390/cancers13174305 (PMC8431118; doi:10.3390/cancers13174305)
Supplement: Supplementary file 1 [file cancers-13-04305-s001.zip › cancers-1322701-supplementary.pdf]

cancers

MDPI

Article

Supplementary Material: The Microenvironment of Small Intestinal Neuroendocrine Tumours Contains Lymphocytes Capable of Recognition and Activation after Expansion

Tobias Hofving [1], Frank Liang [2], Joakim Karlsson [3,4], Ulf Yrlid [2], Jonas A. Nilsson [3,4,*,†], Ola Nilsson [1,†] and Lisa M. Nilsson [3,4]

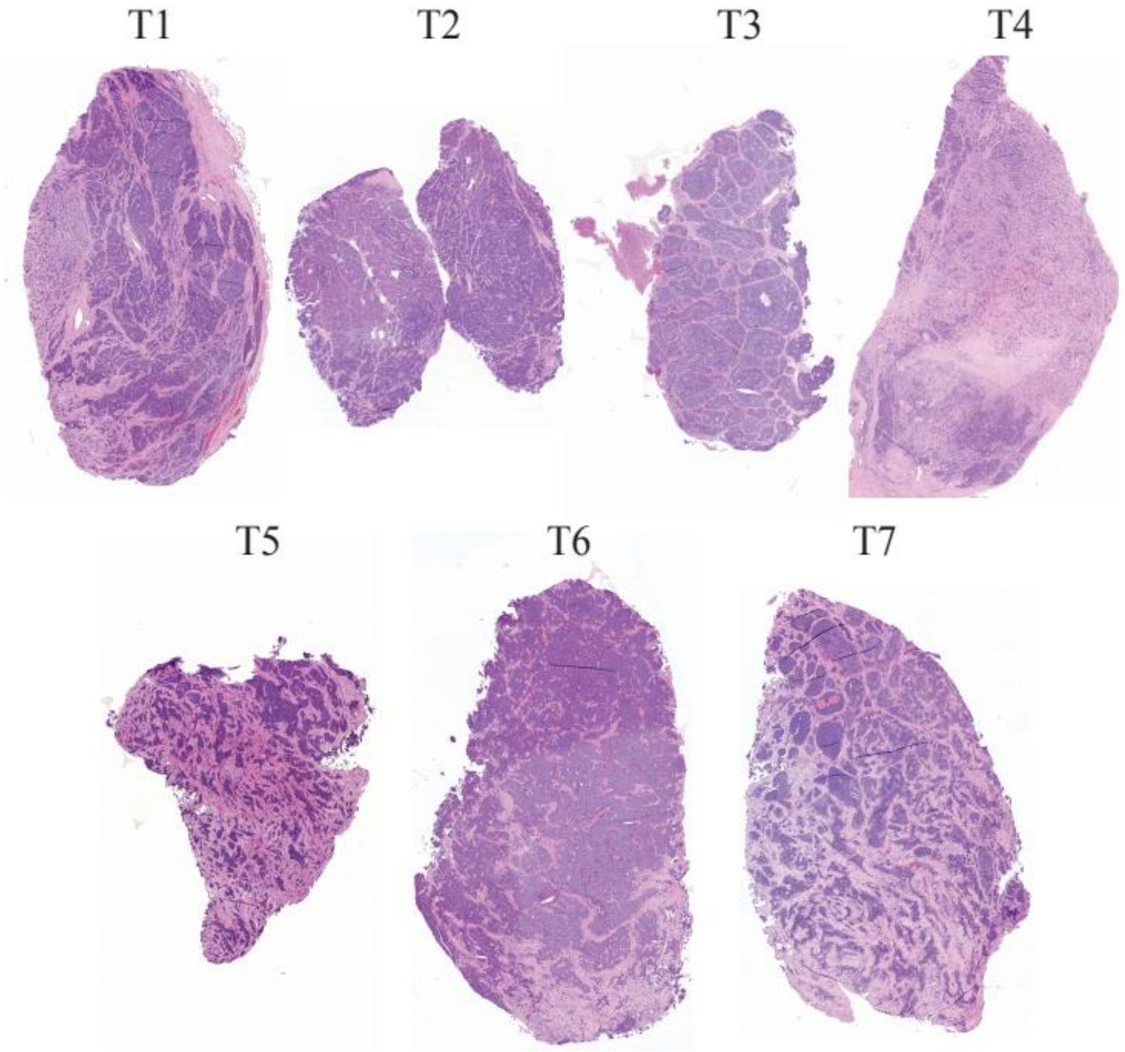

Figure S1. Eosin & haematoxylin (H&E) staining of seven small intestinal neuroendocrine tumours used in the characterisation of the immune landscape. H&E staining showed that the investigated tumours consisted solely of tumour cells and cells of the tumour stroma. No preserved lymph node tissue could be observed.

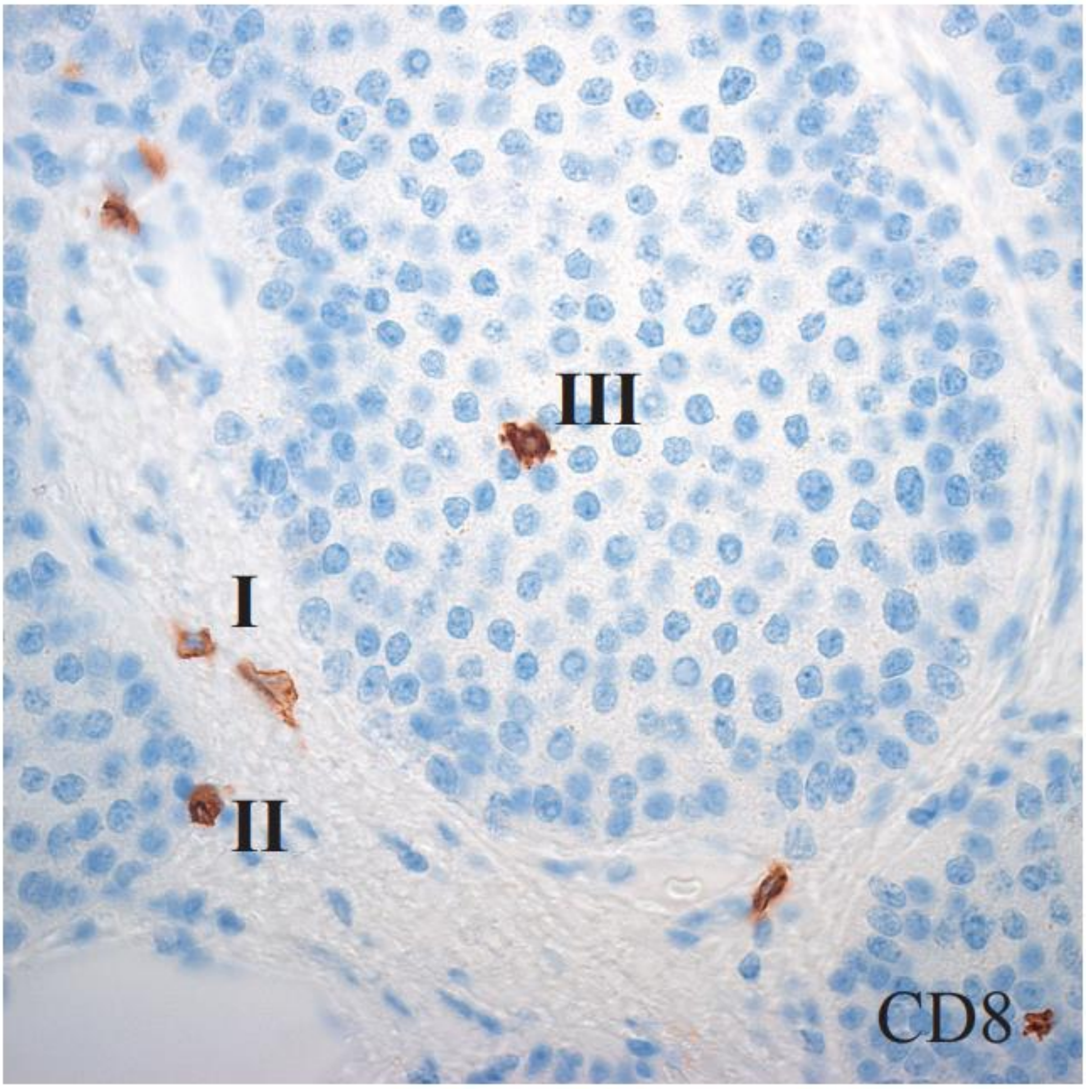

Figure S2. Localisation of CD8+ T lymphocytes. CD8+ T lymphocytes frequently localised to the tumour stroma (I) and the tumour stromal interphase (II), but was also found to infiltrate the tumour nests and reside intra-tumourally (III).

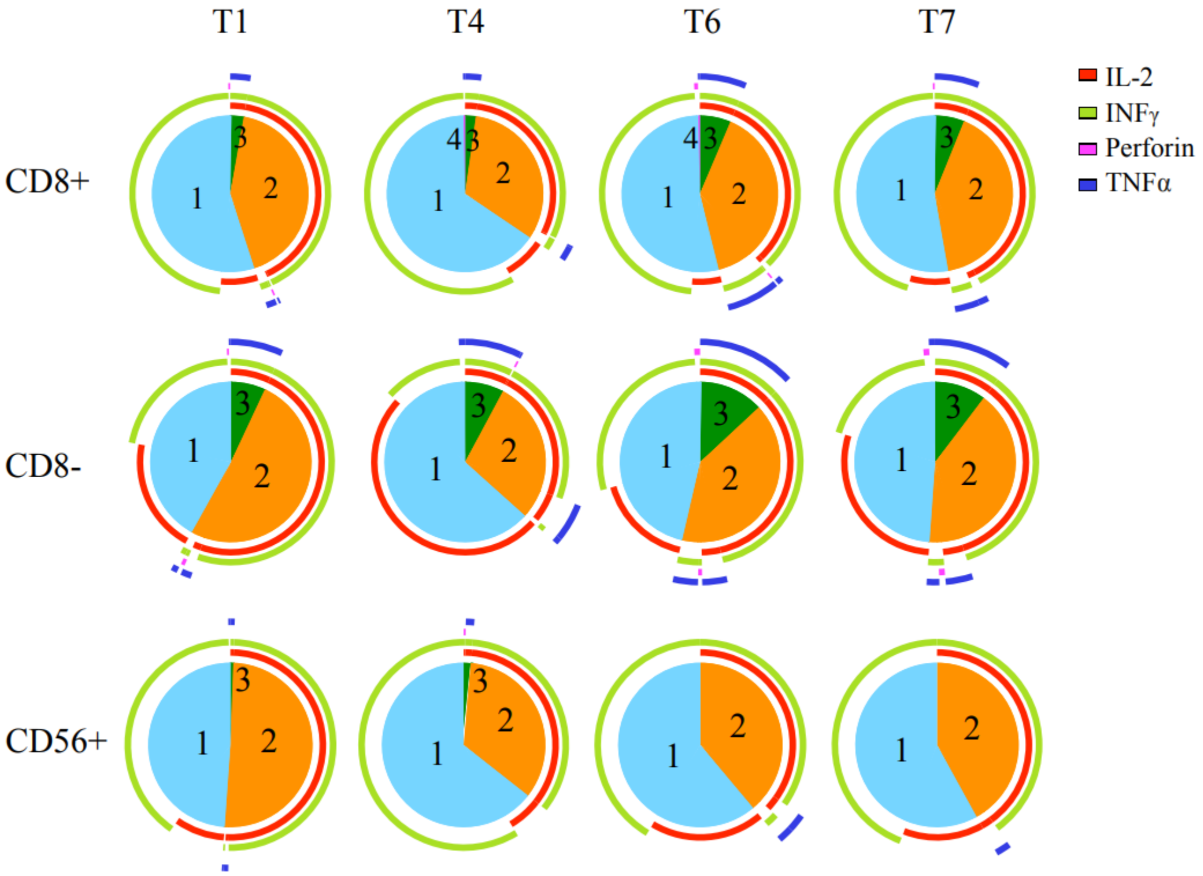

Figure S3. Polyfunctionality of CD8+ T lymphocytes, CD8- lymphocytes, and CD56+ NK cells. Overall, CD4+/CD8 T lymphocytes from tumours T6 and T7 and CD56+ NK-cells from tumour T4 have higher polyfunctionality compared to other tumours. Each pie slice indicates proportion of cells that expressed 1, 2, 3, or 4 different cytokines. Arches represent the proportion of cells that expressed the specific cytokine IL-2, IFNγ, perforin, or TNFα.

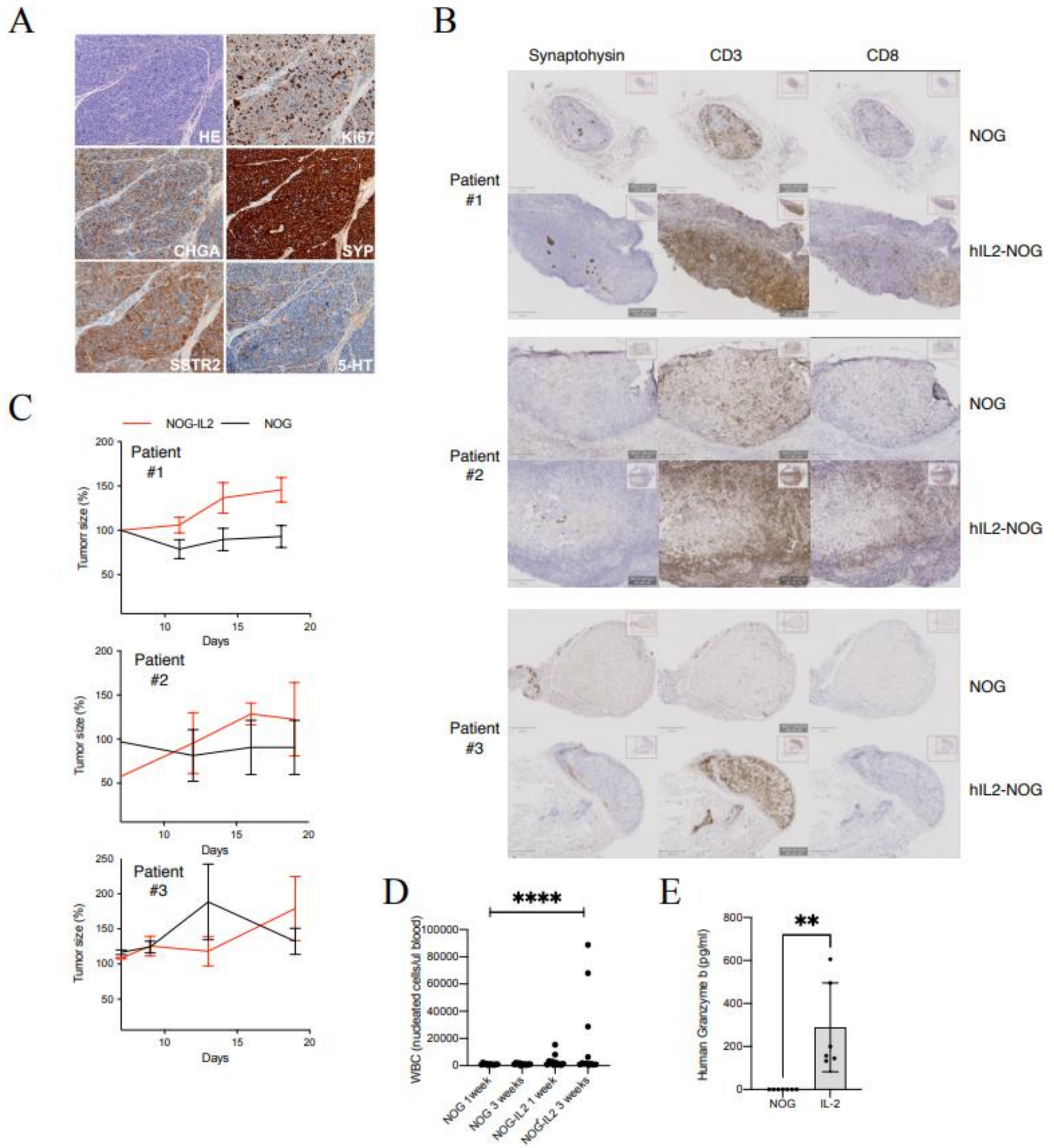

Figure S4. Establishment of small intestinal neuroendocrine tumour patient-derived xenografts. (**A**). One hepatic metastasis from an SINET grew after transplantation to NOG mice. The tumour expressed marker proteins characteristic of SINETs, including chromogranin A (CHGA), synaptophysin (SYP), somatostatin receptor subtype 2 (SSTR2), and serotonin (5-HT). (**B**) Tumour graft transplantations into four NOG or four hIL2-NOG mice per patient sample. Immunohistochemical analysis of transplanted tumours from three different patients three weeks after transplantation using antibodies directed against indicated proteins. Representative images of staining performed on four tumour transplants in each patient are shown. (**C**) Tumour growth was measured using calipers and the growth was established by comparing the size of the first measurements with consecutive measurements. (**D**) White blood cell counts of the mice transplanted with biopsies from three patients. (**E**) Granzyme B measurements using ELISA demonstrating expansion and/or activation of T cells only in hIL2-NOG mice.
